# Endoscopic image-guided laser treatment system based on fiber bundle laser steering

**DOI:** 10.1038/s41598-023-29392-4

**Published:** 2023-02-28

**Authors:** Yuto Miyoshi, Takahiro Nishimura, Yu Shimojo, Keita Okayama, Kunio Awazu

**Affiliations:** 1 Graduate School of Engineering, Osaka University, Yamadaoka 2-1, Suita, Osaka 565-0871 Japan; 2 Graduate School of Medicine, Osaka University, Yamadaoka 2-2, Suita, Osaka 565-0871 Japan; 3 Global Center for Medical Engineering and Informatics, Osaka University, Yamadaoka 2-2, Suita, Osaka 565-0871 Japan

**Keywords:** Biomedical engineering, Biophotonics

## Abstract

A miniaturized endoscopic laser system with laser steering has great potential to expand the application of minimally invasive laser treatment for micro-lesions inside narrow organs. The conventional systems require separate optical paths for endoscopic imaging and laser steering, which limits their application inside narrower organs. Herein, we present a novel endoscopic image-guided laser treatment system with a thin tip that can access inside narrow organs. The system uses a single fiber bundle to simultaneously acquire endoscopic images and modulate the laser-irradiated area. The insertion and operation of the system in a narrow space were demonstrated using an artificial vascular model. Repeated laser steering along set targets demonstrated accurate laser irradiation within a root-mean-square error of 28 $\mu$m, and static repeatability such that the laser irradiation position was controlled within a 12 $\mu$m radius of dispersion about the mean trajectory. Unexpected irradiation on the distal irradiated plane due to fiber bundle crosstalk was reduced by selecting the appropriate laser input diameter. The laser steering trajectory spatially controlled the photothermal effects, vaporization, and coagulation of chicken liver tissue. This novel system achieves minimally invasive endoscopic laser treatment with high lesion-selectivity in narrow organs, such as the peripheral lung and coronary arteries.

## Introduction

The combination of endoscopic access and laser treatment has been successfully used in a wide range of clinical applications, including endoscopic laser tumor resection^1,2^, endoscopic photodynamic therapy^3^, endoscopic photothermal therapy^4,5^, and laser angioplasty^6^. Compared with laparotomy and laparoscopic access, endoscopic access minimizes the number of incisions and sutures^7^. Furthermore, laser treatment is contactless, has high selectivity and high resolution, and has the possibility of automation based on robotic techniques^8^ and numerical simulations^9–11^. Thus, endoscopic laser treatment offers minimally invasive treatment, short surgery time, and optimal precision^7^. The range of endoscopic access has been widely extended by the development of a miniaturized endoscopic tip with high flexibility and functionality^12–14^. For example, the instrument diameter is 12.8 mm for the colonoscope^15^, 3.0 mm for the bronchoscope^16^, and 1.7 mm for the angioscope^17^. The expansion of endoscopic access throughout the body will increase the range of sites in which endoscopic laser treatment can be used.

Effective endoscopic laser treatment requires control of the laser irradiation of target lesions under endoscopic observation. The control of laser irradiation is dependent on the size of the device available for endoscopic use, robustness and accuracy of the laser irradiation position, reproducibility in repeated irradiation, and maximum speed. Several techniques have been proposed for laser steering under endoscopy by “fiber steering”. The distal end of the optical fiber for laser delivery is bent to position the laser spot on the lesion site using magnetic attraction^18–20^, hydraulic pressure^21^, or a tendon-driven manipulator^22^. This fiber steering-based approach requires minimal complex manufacturing and achieves magnetic resonance imaging (MRI)-guided microsurgery. Another approach is based on laser steering by micro electro mechanical system (MEMS) mirrors under endoscopic view^23,24^. The MEMS miniaturization approach achieves both a wide steerable range and high-speed steering. This approach is also applicable for use in conjunction with a colonoscope. Although the devices to be used under an endoscope have diameters ranging from 3-13 mm, two optical channels are required for endoscopic imaging and laser irradiation. This limitation prevents the use of endoscopic laser treatment in narrow organs. For example, although effective laser treatments are already established in the peripheral lung or in a coronary artery, there is no steering device available due to the small size of these organs; the device used in these organs must have a diameter of less than 1 mm for both endoscopic imaging and laser steering^12,25,26^. In addition, there is currently an issue with the alignment of the field of view and the laser irradiation position.

In endoscopic imaging and laser irradiation, devices have been successfully miniaturized using an optical fiber bundle. The optical fiber bundle consists of many transparent cores and claddings. The cores each serve as an optical path. The relative positions of the individual cores on both ends are the same. In fiber bundle imaging, the cores can transfer information about image pixels at each corresponding position. Fiber bundled-based endoscopes miniaturized to less 1 mm have been applied clinically^27,28^. In laser steering, a core on the distal end to the output laser can be selected for the laser irradiation position by inputting laser light to a specific core on the proximal end^29^. Fiber bundle laser steering has been applied in confocal laser fluorescence endoscopy^30^. According to the output core placement, the laser spot is formed via a graded index (GRIN) lens on the distal irradiated plane. Integrating the imaging and laser scanning of the optical fiber bundle will achieve an endoscopic laser treatment system within the size of a single fiber bundle ($< 1$ mm).

Herein, we describe a novel endoscopic laser treatment system based on fiber bundle laser steering that achieves both miniaturization and accurate steering to extend the applications of minimally invasive treatment. The drive mechanism of the fiber bundle laser steering is located on the proximal end, and the distal end design is the same as the fiber bundle imaging system. This allows endoscopic imaging and laser steering to be integrated into a single optical channel via a fiber bundle. Thus, the diameter of the distal end is minimized to the size of the fiber bundle. This article describes the principles of fiber bundle laser steering and investigates the steering capability of a prototype system. Furthermore, the light intensity profile on the distal irradiated plane was evaluated to assess the effect of crosstalk, which is unique to optical transmission using a fiber bundle. This study contributes to the development of a minimally invasive endoscopic laser treatment with a miniaturized laser steering device and thin endoscope. This novel system has improved lesion-selectivity in narrow organs that are difficult to reach with conventional devices.

## Fiber bundle-based laser treatment system

**Figure 1:**
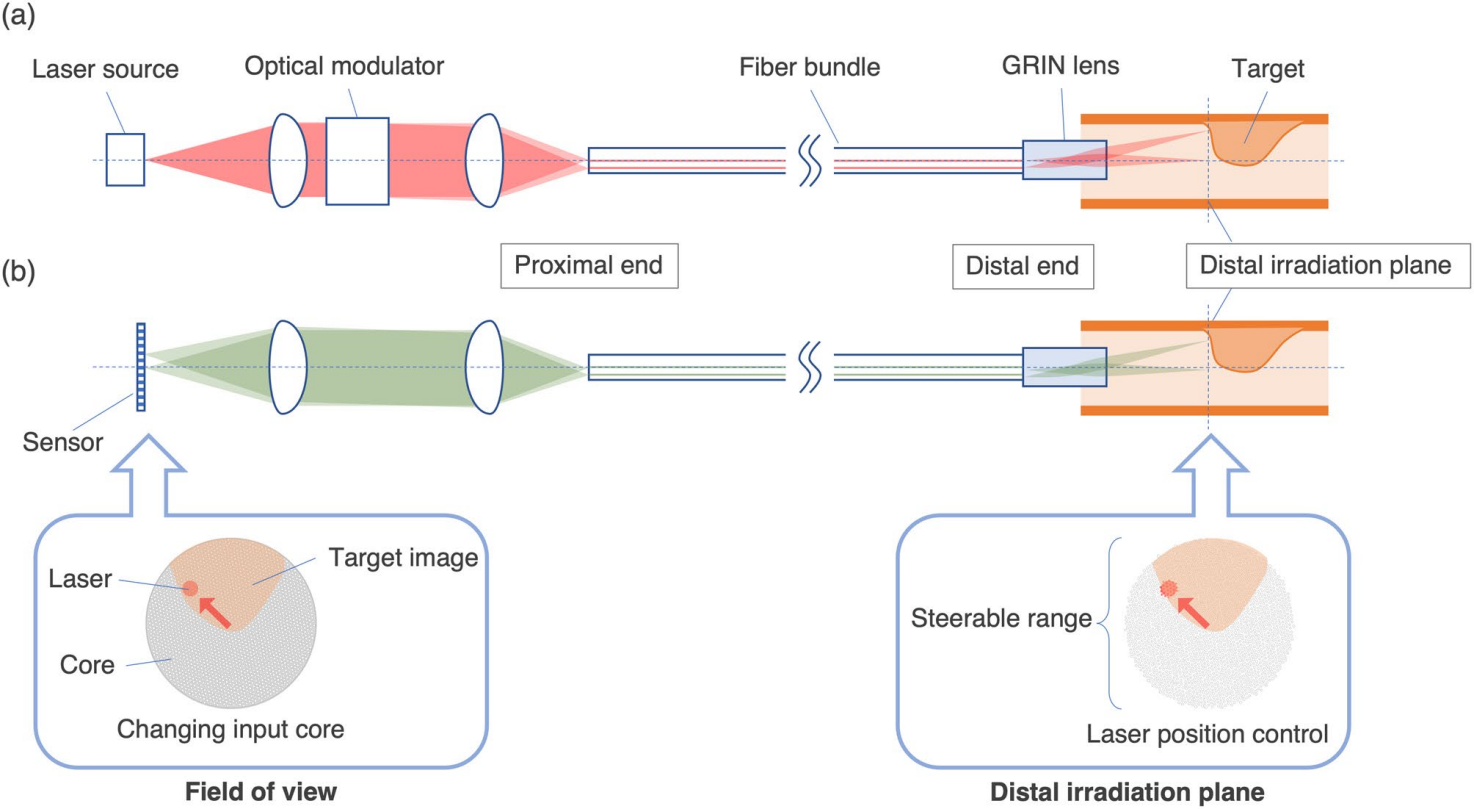
Schematic illustration of our endoscopic image-guided laser treatment system using a fiber bundle for (**a**) laser steering and (**b**) endoscopic imaging. The figure was generated by the authors with Microsoft Power Point for Mac (v. 16.69.1)^35^.

Figure 1 shows a schematic illustration of the novel fiber bundle-based laser treatment system. By inputting laser light into a core on the proximal end, the corresponding core outputs the laser light on the distal end to achieve laser steering^29,31^. The emitted light is imaged by a GRIN lens on the distal irradiated plane to form the laser spot. The position of the spot is controlled by inputting laser light into the corresponding core on the proximal end. Laser steering is achieved by controlling the core placement on the proximal end where the laser is inputted. The steerable range is expressed as the product of the image circle diameter of the fiber bundle and the magnification of the GRIN lens. The steerable range can be extended by using a thicker fiber bundle, which necessitates an increase in the diameter of the device. The maximum steering speed is determined by the drive mechanism placed on the proximal side of the system, and high-speed steering can be achieved using a galvo mirror system. In contrast to other laser steering techniques, this system avoids decreased laser position reproducibility during high-speed steering with miniaturization. By inputting multiple cores, the laser spots for each of the corresponding cores can be formed simultaneously. The basic configuration on the distal side for laser steering is similar to endoscopic imaging via a fiber bundle^27,32–34^. An object on the distal irradiated plane is imaged on the distal end of the fiber bundle, and each core transmits pixel information to the proximal end. By imaging the proximal end with the camera sensor, an image of the distal irradiated plane can be acquired. The field of view is equivalent to the steerable range. Thus, laser steering and endoscopic imaging can be implemented via a single fiber bundle, and the device size is reduced to a diameter of 1 mm or less^27,28^.

The light intensity profile on the distal irradiated plane is a critical factor affecting the laser treatment. The fiber bundle laser steering results in laser crosstalk between cores^36–38^. This might lead to unintentional damage to normal tissue during the laser treatment. The crosstalk between cores is dependent on the core density, shape, and fiber bundle bending conditions, which cause irregular changes in the light intensity profile^39–41^. It is essential to understand and reduce the randomness of the laser intensity profile.

## Methods

### Endoscopic laser treatment system based on fiber bundle laser steering

**Figure 2:**
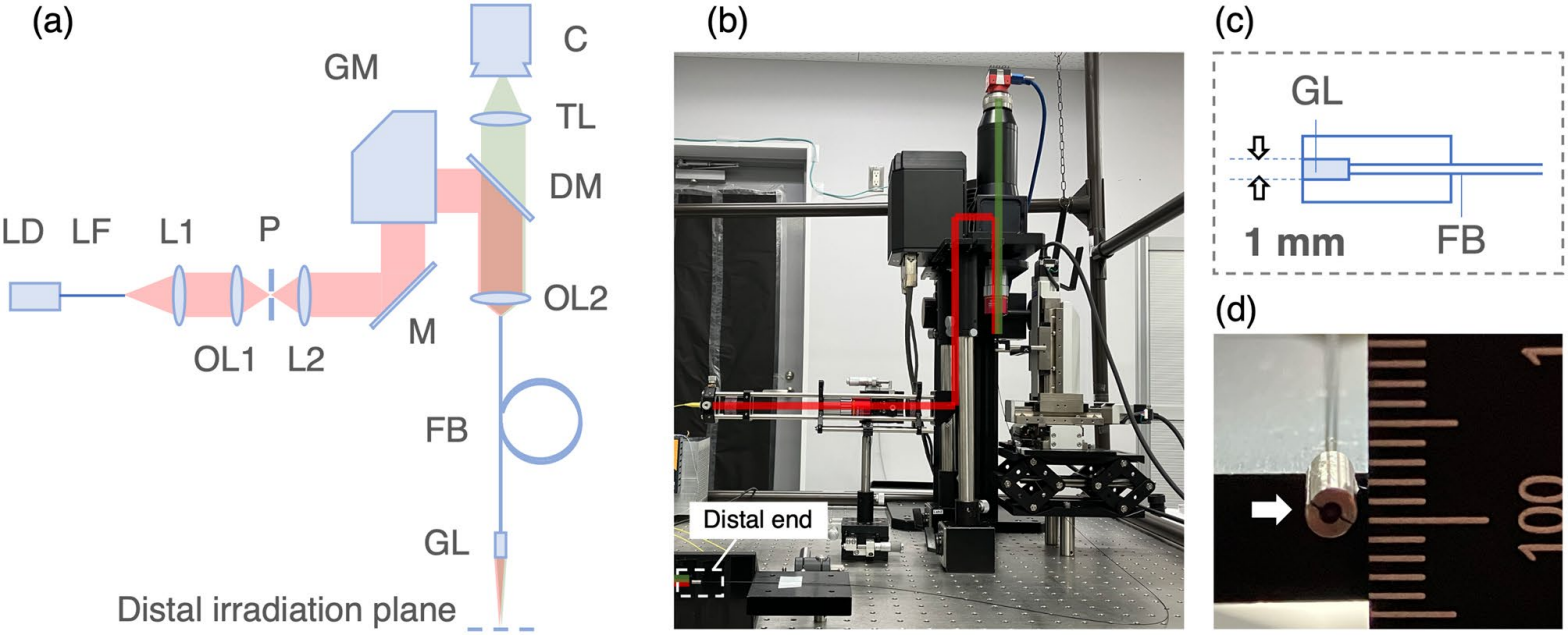
Endoscopic image-guided laser treatment system based on fiber bundle laser steering. (**a**) Schematic illustration of optical setup. The figure was generated by the authors with Microsoft Power Point for Mac (v. 16.69.1)^35^. (**b**) The constructed setup. Red and green lines indicate optical path for laser irradiation and imaging, respectively. (**c**) Internal structure of the distal end. (**d**) External view of the distal end. LD, laser diode; LF, laser fiber; L1, L2, lens; P, pinhole; OL1, OL2, objective lens; M, mirror; GM, galvo mirror system; DM, dichroic mirror; FB, fiber bundle; GL, GRIN lens; C, CMOS camera; TL, tube lens.

A fiber bundle-based laser treatment system was constructed to demonstrate endoscopic laser steering via a single fiber bundle. Figure 2(a) shows a schematic illustration of the optical setup. A fiber bundle (FB; FIGH-17-600G, Fujikura) was prepared to deliver image information for endoscopic imaging and laser energy for treatment. The number of cores was 17,000, and the image circle diameter was 560 $\mu$m. The average core diameter was 2.9 $\mu$m. The numerical aperture (NA) relates to the fiber coupling efficiency. Since the NA of the fiber bundle was undisclosed by the manufacturer, laser input conditions were not optimized. Optimization will be necessary in the future. The distal end of the fiber bundle was combined with a GRIN lens held by a stainless-steel holder having an external diameter of 2.5 mm. Combinations of equipment (LD, LF, L1, OL1, P, L2, OL2, and GL in Fig. 2a) were changed for each evaluation and demonstrated as shown in Table 1. The collimated laser beam from a laser source was passed through the beam expander to adjust the beam diameter and improve the beam quality. The collimated beam for laser treatment was then delivered to a two-axis galvo mirror system (GM; XG210-AG, Thorlabs). The GM was driven by a power supply (GPWR15, Thorlabs) and controlled through a computer on a communication protocol XY2-100. The angle-modulated collimated beam produced by the galvo mirror system was reflected on a short-pass dichroic mirror (DM; DMSP650R, Thorlabs) to combine the imaging optical path. The beam then passed through objective lens (OL2) to be focused onto target cores on the proximal end of the fiber bundle. The transmitted laser was emitted from specific cores at the distal end. The emitted laser was focused onto the distal irradiated plane by a 23.1$\times$ GRIN lens (GL; working distance, 20 mm; GT-IFRL-100-020-50-NC, GRINTech) for laser steering in a narrow space, or 5.86$\times$ GRIN lens (GL; working distance, 5 mm; GT-IFRL-100-005-50-NC, GRINTech) for laser steering in a wide space (Table 1). A CMOS camera (C; Alviumm 1800 U-507, Allied Vision) was used to capture the image of the target plane. A tube lens (TL; focal length, 200 mm; WFA4100, Thorlabs) was used to image the distal irradiated plane onto the CMOS plane.

Figure 2(b) shows the setup of the prototype system. The proximal side of the prototype system consists of general laser devices, galvo mirrors, and a CMOS camera. Miniaturization of the whole system will be possible with the design optimization^42^. The distal end consists of only GRIN lens, fiber bundle, and the lens holder (Fig. 2c). By eliminating the steering mechanism from the distal side in the prototype system, the size of the distal end can be as small as that of the GRIN lens, fiber bundle or holder (Fig. 2d).

**Table 1:** Optical equipment properties of each setup: $\lambda$, laser wavelength; $\phi$, mode field diameter and diameters of the fiber and pinhole; *f*, focal length; *M*, magnification. Section A, Laser steering under endoscopic observation; B, Laser steering evaluation; C, Light intensity profile evaluation; D, Tissue ablation.

	LD: $\lambda$	LF: $\phi$	L1: *f*	OL1: *f*	P: $\phi$	L2: *f*	OL2: *f*	GL: *M*	Section
$\#$1	638 nm	5 $\mu$m	25.4 mm	-	-	-	20 mm	23.1x	A, B
$\#$2	638 nm	5 $\mu$m	25.4 mm	-	-	-	20 mm	5.86x	A
$\#$3	980 nm	5 $\mu$m	25.4 mm	-	-	-	10 mm	23.1x	C
$\#$4	980 nm	5 $\mu$m	25.4 mm	-	-	-	20 mm	23.1x	C
$\#$5	980 nm	106 $\mu$m	25.4 mm	20 mm	10 $\mu$m	16 mm	20 mm	23.1x	C
$\#$6	980 nm	106 $\mu$m	25.4 mm	20 mm	25 $\mu$m	16 mm	20 mm	23.1x	C
$\#$7	980 nm	106 $\mu$m	25.4 mm	-	-	-	20 mm	23.1x	C
$\#$8	980 nm	106 $\mu$m	25.4 mm	20 mm	25 $\mu$m	16 mm	20 mm	5.86x	D
$\#$9	980 nm	106 $\mu$m	25.4 mm	-	-	-	20 mm	5.86x	D

### Laser steering under endoscopic observation

To assess the simultaneous performance of imaging and laser steering by the endoscopic laser treatment system, a target pattern printed on white paper was placed on the distal irradiated plane. A red laser was used for visualizing the laser irradiation position with setup $\#$1. The laser input position into the core was controlled by the galvo mirror system, and the irradiation position on the distal irradiated plane was observed. To demonstrate the laser steering in a narrow space, the distal end of the system was inserted into a vascular model with an atherosclerotic plaque (diameter, 8 mm; HXBVX01P08009JX, WetLab) with setup $\#$2. The inside of the model was illuminated with a light-emitting diode (SOLIS-3C, Thorlabs) from the outside. The model of the plaque was colored with black ink to make it easier to distinguish from the vascular wall model and to observe the movement of the laser spot on the target.

### Laser steering evaluation

The precision and accuracy of laser steering were evaluated using three target patterns: a circle, a wavy circle, and a four-leaved pattern. The target patterns were printed within a circle with a diameter of 4.3 mm. A beam profiler (sensor size, 6.7 $\mu$m; frame rate, 16 fps; LaserCam-HR, Coherent) was placed at the distal irradiated plane to continuously record the light intensity profile during laser steering. The laser steering was conducted five times along each target pattern by automatic modulation of the galvo mirror system with setup $\#$1. The steering speed was 0.17 mm/s on the distal irradiated plane. The laser irradiation position was defined as the center of gravity of the light intensity profile. The accuracy, which represents the performance of the laser irradiation position control with respect to the target, was evaluated as the mean of the root-mean-square error (RMSE) between the target position and the irradiation position. The precision, which represents the reproducibility of the laser irradiation position control, was evaluated using the standard deviation of the RMSE.

### Light intensity profile evaluation

To characterize the effect of crosstalk between cores, the light intensity profiles on the distal irradiated plane were observed by changing the input laser diameter on the proximal end of the fiber bundle. In addition to evaluating the laser input into a single core, the simultaneous laser inputs on multiple cores were evaluated by expanding the laser spots at the proximal ends of the cores. The spot sizes for inputting the core(s) were adjusted from 5.0 to 81.9 $\mu$m with the optical setups $\#$3, 4, 5, 6 and 7 shown in Table 1. A beam profiler was placed on the distal irradiated plane, and 100 images of the profile were taken for each optical setup while the laser input position on the proximal end was randomly changed.

The randomness of the profile on the irradiation position was evaluated. A region of interest (ROI) was extracted from the image; the center of the ROI was the center of the gravity of the laser intensity profile. The number of pixels per side of the ROI, *R*, was defined as $R = r M / l + 2C$, where *r*, *M*, *I*, and *C* were the input diameter, the magnification of the GRIN lens, the pixel size of the beam profiler, and the measured maximum distance from the center core to the crosstalk core (in pixels), respectively. The maximum distance was 125 pixels when the laser was inputted into a single core. The gray values were cumulatively summed from the center of the ROI to the outside concentrically and were then normalized by the total gray value in the ROI, defined as normalized accumulative gray value (NAGV). Furthermore, a diameter of the concentric circles satisfying NAGV = 0.5, defined as 50$\%$ diameter, was calculated. The 50$\%$ diameter was calculated for all images and statistically analyzed for each optical setup.

### Tissue ablation

Tissue ablation tests were performed with setup $\#$8 and $\#$9 to demonstrate the control of the photothermal effect position on tissue. Commercially available chicken liver was used as a sample. Frozen chicken livers were cut into 5-mm-thick pieces and thawed. The sample was placed vertically on the distal irradiated plane. The power density of the irradiated laser was 10–30 W/mm$^2$ at the sample plane. The steering speed was 0.1 mm/s on the distal irradiated plane. For irradiation on a fixed point, the irradiation time was about 2 seconds.

## Results

### Laser steering under endoscopic observation

**Figure 3:**
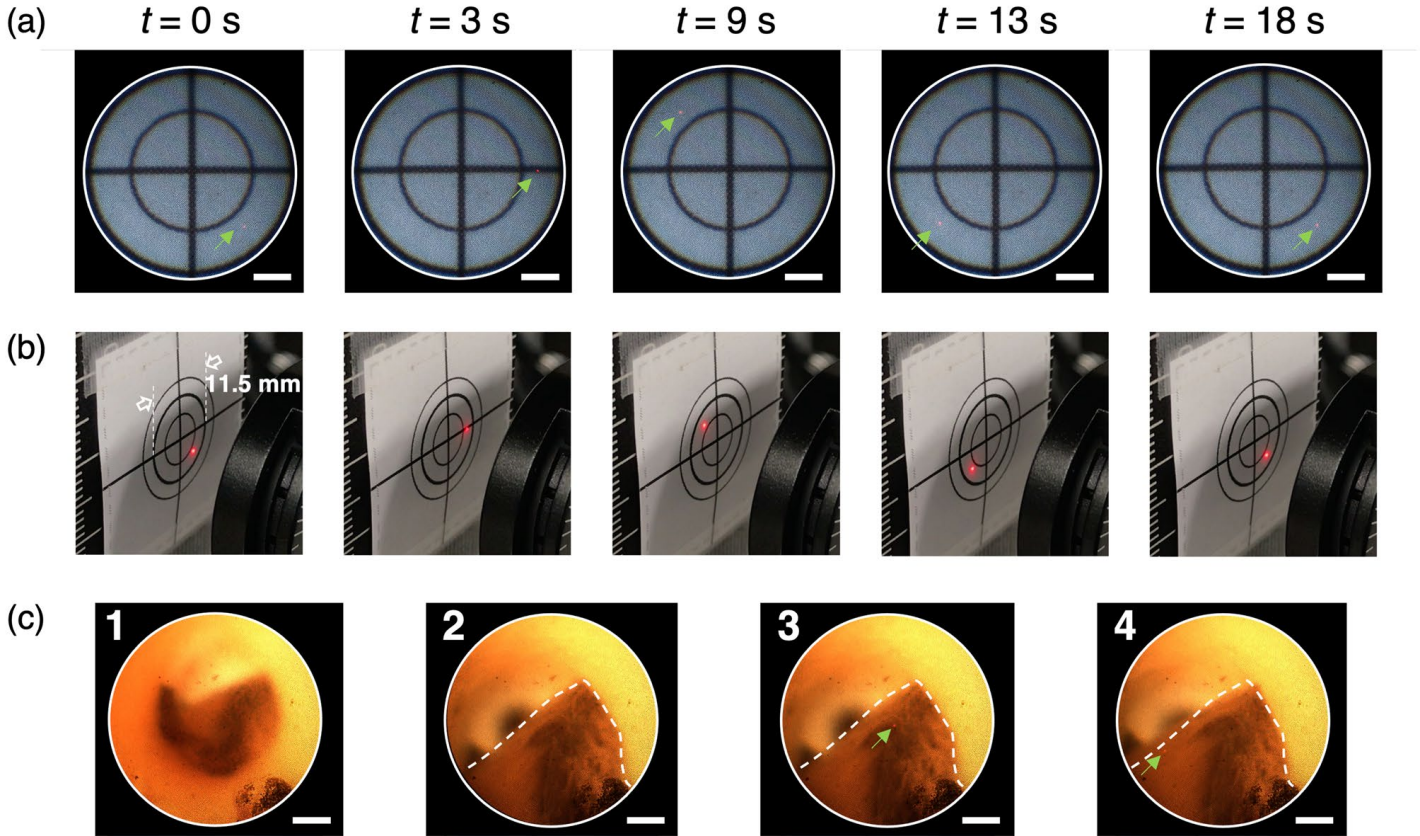
(**a**) Snapshot of the endoscopic view in the laser steering demonstration (Supplementary Movie S1). The green arrows indicate the laser input position on the proximal end. Scale bar: 100 $\mu$m. (**b**) Snapshot of the external view of the distal end. (**c**) Laser steering demonstration using a vascular model with black-colored plaque (Supplementary Movie S2). The white dotted line indicates the plaque. The green arrows indicate the laser input position on the proximal end. Scale bar: 100 $\mu$m.

Figures 3(a,b) show the snapshots of the endoscopic and external views during laser steering on the target pattern. The time course of the snapshots is shown in Supplementary Movie S1. The implemented system can move the irradiation position on the distal irradiated plane by controlling the input position using the galvo mirror system.

Figure 3(c) shows the snapshots of the endoscopic view during laser steering in the vascular model. The Supplementary Movie S2 shows its time course. The distal side of the system was easily inserted into the model, and the endoscopic view was successfully acquired in real time. The irradiation position was controlled under image guidance, which enabled the laser light to be focused on the plaque area in the lumen without exposing the vascular wall. The field of view and the steerable area on the distal irradiated plane were identical, which means that the object visibility and the irradiation position were synchronized, and that intuitive steering was possible.

### Laser steering evaluation

**Figure 4:**
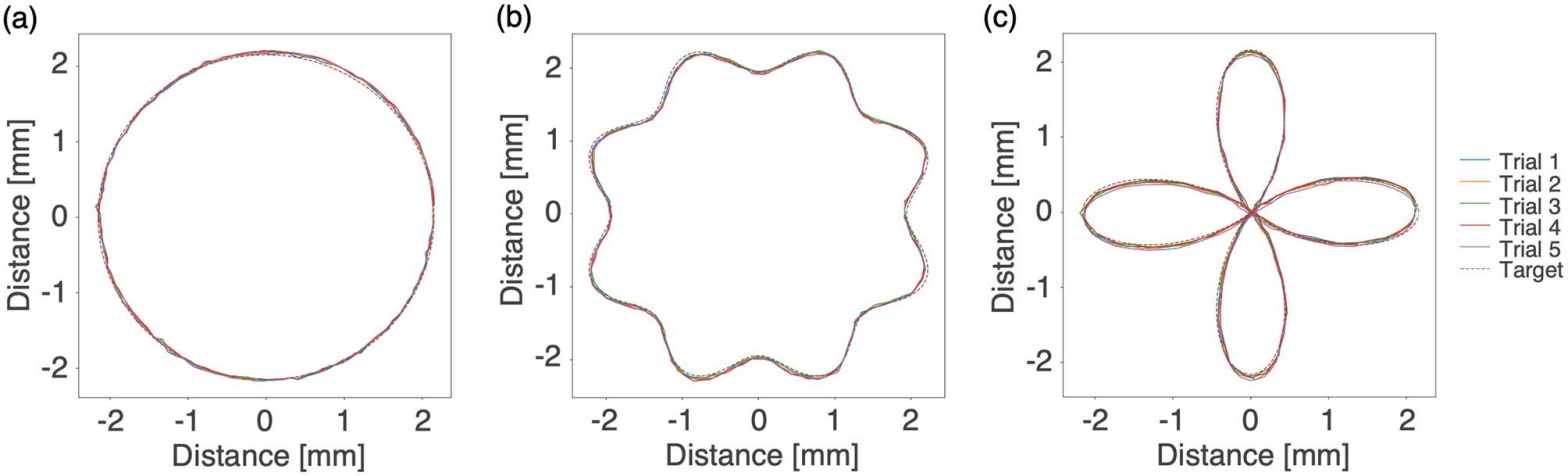
Laser spot trajectories for (**a**) circle, (**b**) wavy circle, and (**c**) four-leaved targets.

To evaluate the laser steering controllability and repeatability, the laser steering trajectories along 2D targets on the distal irradiated plane were repeatedly measured (Fig. 4). The accuracy in laser steering to targets with a circle, wavy circle, and four-leaved pattern was 27, 28, and 27 $\mu$m, respectively. Even in the case of the four-leaved target that had complex trajectories requiring abrupt changes in direction, the accuracy of the laser steering showed little change. The precision in laser steering to the circle, wavy circle, and four-leaved targets were 4, 4, and 6 $\mu$m, respectively. The maximum 2$\sigma$ ($\sigma$: the standard deviation) was 12 $\mu$m in the case of the four-leaved target, which means that 95$\%$ of the laser irradiation position was controlled within a 12 $\mu$m radius of dispersion about the mean trajectory.

### Light intensity profile evaluation

**Figure 5:**
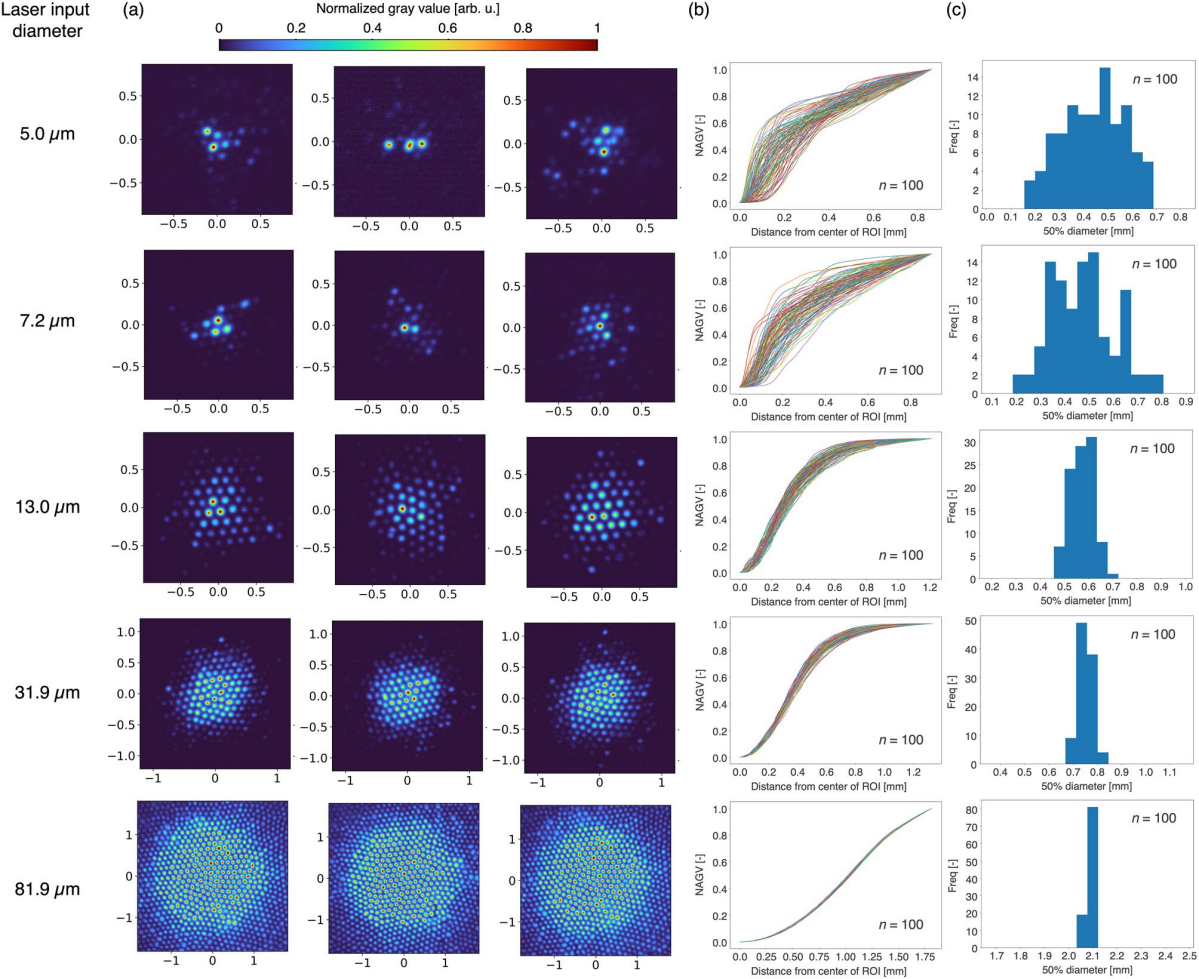
(**a**) Light intensity distribution examples on the distal irradiated plane for the input laser diameters of 5.0, 7.2, 13.0, 31.9, and 81.9 $\mu$m. All images were normalized by the maximum gray value in each image (unit: mm). (**b**) NAGV at various distances from the center of ROI. (**c**) Histograms of the diameter of the circle including 50$\%$ of the total gray value in the region of interest.

Figure 5(a) and Supplementary Fig. S1 show three examples of the light intensity profile on the distal irradiated plane created by inputting laser from a randomly selected position on the proximal end for the diameters of 5.0, 7.2, 13.0, 31.9, and 81.9 $\mu$m with setups $\#$3, 4, 5, 6, and 7. The laser spots on the distal irradiated plane were distributed randomly by moving the laser input position on the proximal end. The presence of core-to-core crosstalk was confirmed. The smaller laser input diameter created a discrete spot on the distal irradiated plane. For the input diameter of 5.0 $\mu$m, which was the smallest in this experiment, a region of high gray values sometimes appeared far from the center. For the input diameters of 13.0, 31.9, and 81.9 $\mu$m, the light intensity profiles approached a circular shape, which was the same as the laser projection shape on the proximal end.

Figure 5(b) shows the relationship between the distance from the center of ROI and the NAGV. The increase in the NAGV was not uniform for the input diameters of 5.0 and 7.2 $\mu$m. This means that spots with high gray values could emerge far from the center, and their locations were random regardless of the input position. For the input diameters of 13.0, 31.9, and 81.9 $\mu$m, the increase in the NAGV showed a constant pattern.

**Figure 6:**
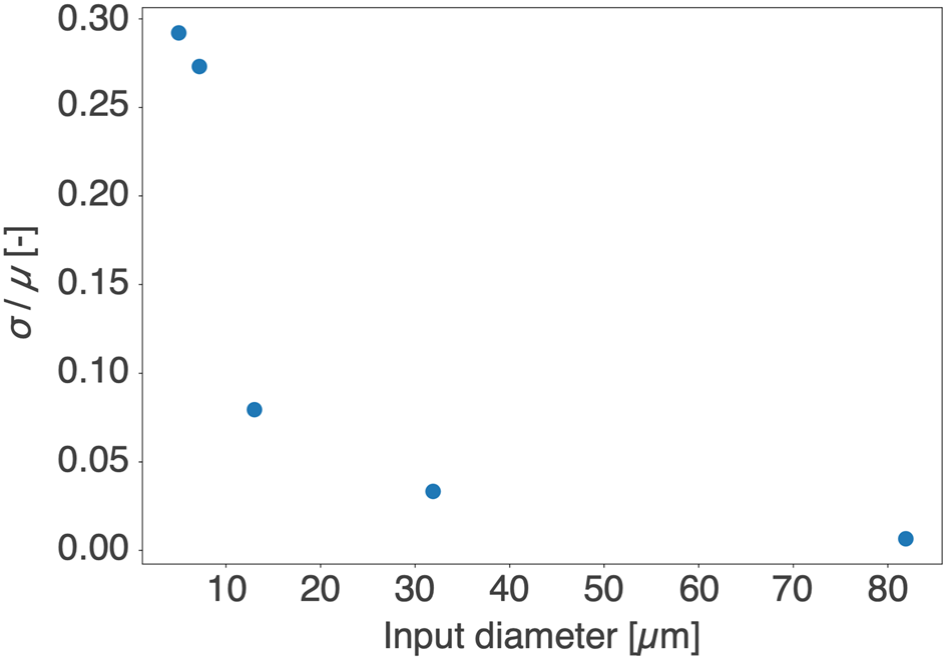
Standard deviation $\sigma$ divided by the average $\mu$ of the 50$\%$ diameter.

Figure 5(c) shows histograms of the 50$\%$ diameter. The values were 0.44 ± 0.13, 0.47 ± 0.13, 0.57 ± 0.05, 0.75 ± 0.03, and 2.083 ± 0.014 mm for the input diameters of 5.0, 7.2, 13.0, 31.9, and 81.9 $\mu$m. The mean of the 50$\%$ diameter tended to increase as the input diameter increased. The standard deviation of the 50$\%$ diameter tended to decrease as the input diameter increased. The standard deviation normalized by the mean of the 50$\%$ diameter decreased as the input diameter increased (Fig. 6). Based on these results, we concluded that the randomness of the spot locations due to crosstalk was reduced by inputting into multiple cores.

### Tissue ablation

**Figure 7:**
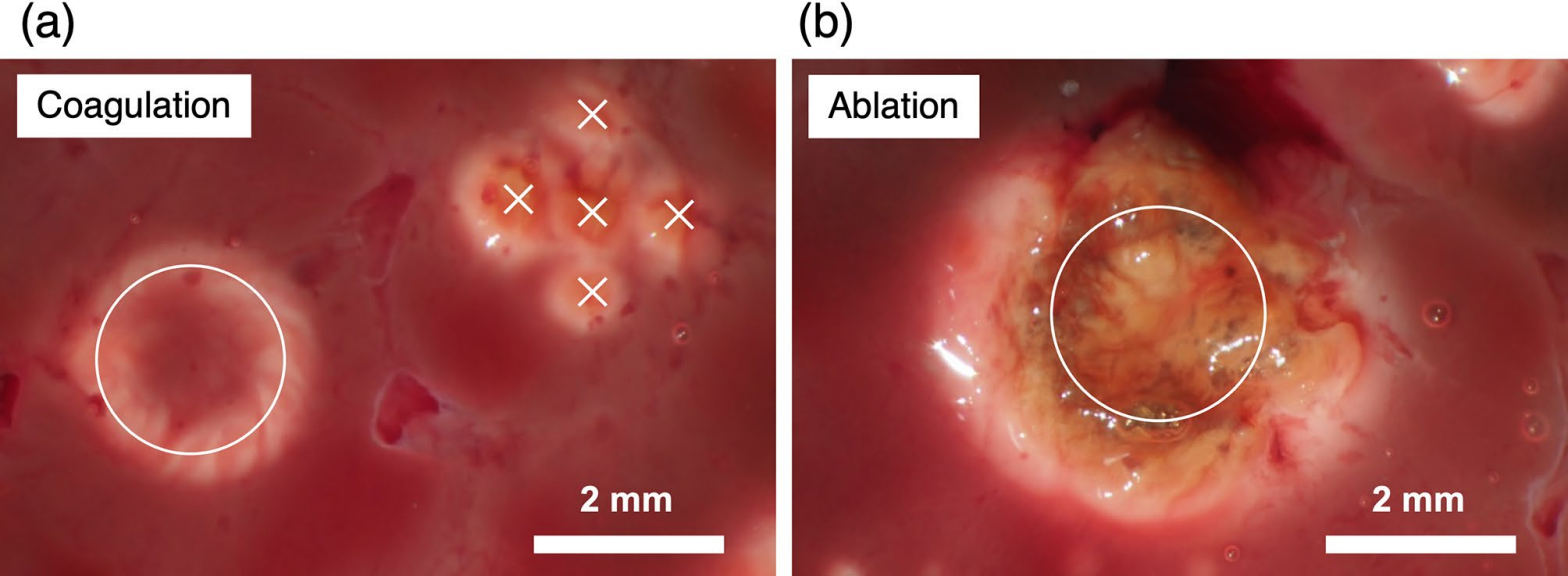
Photographs of chicken liver after (**a**) tissue coagulation and (**b**) ablation. The white circles and crosses indicate the laser steering trajectories and irradiated points, respectively.

To demonstrate the potential of the system for use in laser treatment, we tested the ability of the system to perform tissue coagulation and ablation. Figure 7(a) shows coagulated tissue after laser irradiation with setup $\#$8. The power density on the sample plane was 10.7 W/mm$^2$. Tissue coagulation was observed at five irradiated points and in a circular pattern along the laser steering trajectory. This implies that the system has the capability to control the photothermal effect position on the tissue under endoscopic observation. Figure 7(b) shows ablated tissue after laser irradiation with setup $\#$9. The power density on the sample plane was 29.1 W/mm$^2$. With a high-powered density, ablation was observed along the circular trajectory. The center of the circle could not be preserved as normal tissue without degeneration due to thermal diffusion into the surrounding areas. The continuous-wave laser irradiation resulted in heat conduction and contributed to excessive collateral thermal damage.

## Discussion

This study demonstrated the effectiveness of a novel miniaturized endoscopic image-guided laser treatment system with laser steering. The irradiation position on the distal irradiated plane is controlled by modulating the laser input position at the proximal end. As the mechanics for laser steering can be implemented on the proximal side, there is no size limitation to place small complex components and to ensure robustness, unlike with fiber steering^18–22^ or MEMS approaches^23,24^ (Fig. 2d). The optics at the distal end are shared with fiber bundle imaging, which enables the optical channel for imaging and laser steering to be combined into a single fiber bundle. In the prototype system, a 1-mm-diameter GRIN lens is fixed to the FB using a 2.5-mm-diameter lens holder. The optical configuration of the distal end was similar to that of a conventional fiber bundle-based endoscope. Diameter sizes less than 1 mm have been achieved with the conventional fiber bundle-based endoscope^27,28^. Similar to the references, the diameter size of the distal end can be reduced to less than 1 mm if necessary. This fiber bundle-based approach may contribute to expanding the application of minimally invasive endoscopic laser treatment to narrow organs that are inaccessible with conventional devices. The system can also be applied to organs that are accessible with conventional devices, such as the pharynx^21^ and colon^23^, while providing a wider spatial margin on the endoscopic tip and enhancing its functions. In addition, laser treatment with inserted fibers without an endoscopic view will be improved by introducing the endoscopic view^43^.

One possible application of the fiber-bundle-based system is in minimally invasive laser angioplasty, which is clinically used for acute myocardial infarction with atherosclerotic plaques^44^. The proposed system will allow for image-guided laser surgery. There is also potential for application in photodynamic therapy of the peripheral lung. Photosensitizing drugs are chemically modified to effectively accumulate in tumors, but can spread to normal tissue^45^. By applying this novel system to a bronchoscope, selective irradiation achieved by fiber bundle laser steering could reduce undesired exposure of reactive oxygen species to normal tissue. Furthermore, this system could allow minimally invasive treatment in pediatric patients who have smaller organs than adults^46,47^. This would reduce the need for laparotomy, which results in a large scar. It is essential to select the laser wavelength depending on the treatment target and method, as the tissue properties of optical scattering and absorption affect the energy deposition^48^. The wavelength for laser treatment can be selected from the wavelength band covered by the optical setup in Fig. 2(a). For the evaluation of the laser-induced thermal effects, a 980-nm-wavelength laser was used in this study. Optimization of the wavelength for treatment is necessary in the future. The wavelength for effective laser-tissue interactions depends on the optical properties of the target tissue. As an instance, laser ablation of bladder soft tissue using wavelengths of 450 or 532 nm has been demonstrated^49^, and these wavelengths are applicable to the proposed system.

Although the image-guided laser steering was achieved, the steering performance was limited due to the principles of this system. The laser propagation in the fiber bundle is dependent only on the core, without any contribution from the cladding. The steerable position on the distal irradiated plane is limited to the position corresponding to the core placement. The irradiated laser light was delivered to the target position via the corresponding core that could create the laser spot nearest to the target position. The core arrangement was parallel but random. This might have caused the slight difference between the targeted and irradiation positions shown in Fig. 4. Despite this natural limitation, the accuracies and precisions of the laser steering were comparable to those reported in previous studies^19,21,24^. In steering with the conventional devices, the miniaturization of the drive mechanism mounted on the distal side of the endoscope results in manufacturing uncertainties and nonlinearities during operation. In our system, these difficulties are avoided by using a galvo mirror system placed on the proximal side of the endoscope to control the irradiation position.

Crosstalk in a fiber bundle is strongly related to treatment resolution and is technically unavoidable (Fig. 5a). This crosstalk is also a challenge in high-quality imaging using a fiber bundle, and this issue has been widely studied^36–38^. As the crosstalk occurs randomly between cores depending on a large number of irregularly shaped cores and the fiber bending state, it is unrealistic to try and estimate the laser leakage^39–41^. Although increasing the core size might reduce crosstalk^50^, the image resolution would decrease. The specifications of the fiber bundle, including the core size, need to be optimized from the point of view of the image and treatment resolutions required by the treatment target. In the present study, we simultaneously tested laser inputting into a single core and into multiple cores. As shown in Fig. 6, the relative variance in the 50$\%$ diameter was decreased by increasing the input diameter. This is probably because superposition of the laser leakage makes the probabilities of spot emergence stable, which results in a reduction of the randomness in the spot location. The fluctuation in power density may be suppressed to avoid laser interactions in the undesired area. In other words, the larger input diameter means that the treatment resolution must be sacrificed in order to suppress the negative effects of crosstalk. This trade-off between the crosstalk effect and the size of the treatment target must be considered.

In the spatial distributions of the irradiated laser intensity on a target (Figs. 5 and S1), the centers of the laser spots are positioned along the arrangements of the cores on the distal end of the fiber bundle. The areas between the neighboring laser spots on the distal irradiated plane are considered as dead areas, as these areas cannot be irradiated by the prototype system. The characteristic patterns reflected on the core arrangement, however, were not found in the spatial distributions of the thermal effects (Fig. 7). This result indicates that the optical and thermal diffusion in the tissue caused the thermal effects in dead area. Thus, there were no incomplete laser treatment area in our result. Since the optical and thermal diffusion depend on laser wavelength, pulse width, and tissue properties, depending on these parameters, the dead area may cause an incomplete laser treatment area.

The tissue ablation experiment demonstrated the ability of the system to control the position of the photothermal effect on tissue (Fig. 7). Tissue coagulation was observed at the laser irradiation position, which implies an improvement in the lesion-selectivity of endoscopic laser treatment (including applications to photochemical therapy and photodynamic therapy), despite the effect differences. Increasing the power density caused tissue ablation, but also caused thermal damage to the surrounding normal tissue. For management of such collateral thermal damage, appropriate laser wavelength and pulse width should be considered in addition to the image-guided laser steering. The laser wavelength can be determined from consideration of lesion area and light propagation in tissue^48^. The pulse width will be designed from thermal confinement condition of target lesions^11^.

## Conclusion

In conclusion, the miniaturized endoscopic laser treatment system using a fiber bundle simultaneously achieved field of view acquisition and laser steering in a vascular model. Repeated laser steering along the targets showed that the accuracy and precision of the system were comparable to the results of previous studies investigating miniaturized MEMS devices^23^ or fiber steering approaches^19^. Evaluation of the light intensity profile showed that the randomness caused by the core-to-core crosstalk was suppressed by increasing the laser input diameter on the proximal end of the fiber bundle. In future image-guided laser treatment, the optical setup needs to be optimized to suit the target lesion by considering the trade-off between the fiber bundle crosstalk and treatment resolution. The fiber bundle laser steering controlled the photothermal effect position on the tissue. Our approach provides a minimally invasive endoscopic laser treatment with image guidance in a lumen with a diameter of less than 1 mm.

## Supplementary Information

Supplementary Information 1.

Supplementary Information 2.

Supplementary Information 3.

## Data Availability

The datasets used and/or analysed during the current study available from the corresponding author on reasonable request.
