# Supplementary material for: Endoscopic image-guided laser treatment system based on fiber bundle laser steering
Source: Sci Rep. 2023 Feb 28;13:2921. doi: 10.1038/s41598-023-29392-4 (PMC9975189; doi:10.1038/s41598-023-29392-4)
Supplement: Supplementary file 3 — Supplementary Information 3. [file 41598_2023_29392_MOESM3_ESM.pdf]

Supplemental Information

Endoscopic image-guided laser treatment system based on fiber bundle laser steering

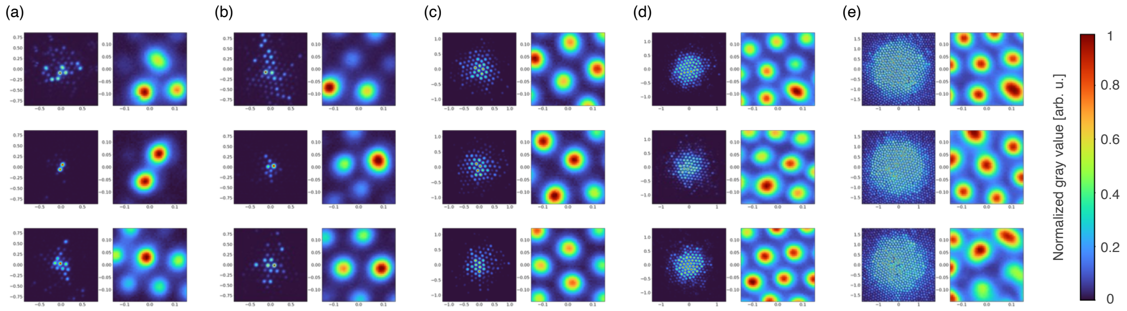

Supplementary Figure S1. Examples of light intensity distributions acquired by optical setups (a) #3, (b) #4, (c) #5, (d) #6, and (e) #7. Each left figure shows light intensity distribution of the ROI and the right figure shows the enlarged image of the middle region of the ROI (unit: mm).
